# Establishing an EU-China consortium on traditional Chinese medicine research

**DOI:** 10.1186/1749-8546-5-42

**Published:** 2010-12-14

**Authors:** Halil Uzuner, Tai-Ping Fan, Alberto Dias, De-an Guo, Hani S El-Nezami, Qihe Xu

**Affiliations:** 1 Department of Renal Medicine, King's College London, London, UK; 2 Department of Pharmacology, University of Cambridge, Cambridge, UK; 3 CITAB-UM, Department of Biology, University of Minho, Braga, Portugal; 4 Shanghai Research Centre for TCM Modernization, Shanghai Institute of Materia Medica, Chinese Academy of Sciences, Shanghai, China; 5 School of Biological Sciences, University of Hong Kong, Hong Kong, China

## Abstract

Traditional Chinese medicine (TCM) is widely used in the European Union (EU) and attracts intense research interests from European scientists. As an emerging area in Europe, TCM research requires collaboration and coordination of actions. Good Practice in Traditional Chinese Medicine Research in the Post-genomic Era, also known as GP-TCM, is the first ever EU-funded 7^th ^Framework Programme (FP7) coordination action, aiming to inform the best practice and harmonise research on the safety and efficacy of TCM through interdisciplinary exchange of experience and expertise among clinicians and scientists. With its increasingly large pool of expertise across 19 countries including 13 EU member states, Australia, Canada, China, Norway, Thailand and the USA, the consortium provides forums and collaboration platforms on quality control, extraction technology, component analysis, toxicology, pharmacology and regulatory issues of Chinese herbal medicine (CHM), as well as on acupuncture studies, with a particular emphasis on the application of a functional genomics approach. The project officially started in May 2009 and by the time of its conclusion in April 2012 a Europe-based academic society dedicated to TCM research will be founded to carry on the mission of GP-TCM.

## Introduction

Traditional Chinese medicine (TCM), especially Chinese herbal medicine (CHM) and acupuncture, is an ancient medical system used in China and other Asian countries for thousands of years [1,2]. In contrast to the reductionist approach of Western medicine based on modern anatomy, physiology, pathology, pharmacology as well as cell and molecular biology, TCM uses a unique system and an individualised and holistic approach to describe health and disease, based on the philosophy of Yin-Yang balance and an emphasis on harmony of functions. These two medical systems differ greatly in many respects. In the past seven years, a number of international organisations were established in mainland China, Hong Kong and Macao, including the World Federation of Chinese Medicine Societies (WFCMS, September 2003), the Consortium for Globalisation of Chinese Medicine (CGCM, December 2003) and the International Society for Chinese Medicine (ISCM, 2004).

The Good Practice in Traditional Chinese Medicine Research in the Post-genomic Era (GP-TCM) consortium was launched by the European Commission on the 1^st ^May 2009. This is a three-year coordination action project funded under the EU Seventh Framework Programme (FP7) with a total budget of €995,100. The central hypothesis of the consortium is that, using functional genomics technology, which allows high-content observations of whole profiles of molecules at different levels, *eg *DNA, mRNA, protein and metabolites, and furthermore linking them to clinically relevant biological functions, we might be in a better position than ever before to interpret and validate the scientific value of TCM in a holistic and function-oriented manner [3-11].

### Objectives

Focusing on research of CHM and acupuncture, we especially emphasise studies of CHMs, their complex chemical ingredients and their holistic impact on the functional genomics of patients. The overall aim of the consortium is to inform the best practice and harmonise research on the safety and efficacy of TCM using a functional genomics approach through exchange of opinions, experience and expertise among scientists in EU member states, China and other parts of the world. Specifically, we aim to undertake the following objectives:

• Develop a European-Chinese network, collaborating on functional genomics research of TCM;

• Review current practice of TCM research, identify problems and propose solutions;

• Propose standard protocols of methodology;

• Propose priority areas for future research;

• Develop online resources to support and enhance pan-European studies of TCM research;

• Facilitate and foster a sustainable European collaboration by founding a European society dedicated to TCM research.

### Structure

**Figure 1:**
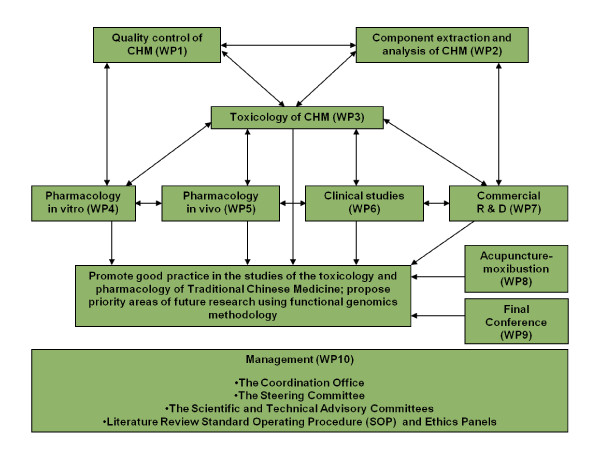
**Structure of the GP-TCM Consortium**. GP-TCM work package interaction and relationship: GP-TCM has ten interactive working groups, known as work packages (WP). WP1-WP7 specialises in quality control, component extraction and chemical analysis, toxicology, *in vitro *and *in vivo *pharmacology, clinical studies and regulatory issues in commercial R&D respectively. WP8 is specialised in acupuncture studies and WP9 is dedicated to organising the Final conference of the consortium, at which a new European society will be launched to succeed the mission of the consortium. WP10 is the managing, coordinating and leading body of the whole consortium, aiming at integrating the expertise and collating outputs of all WPs to achieve the overall objectives of the consortium.

As shown in Figure 1 through ten working groups known as work packages (WPs), the consortium takes actions to review the techniques, identify problems and solutions in the quality control (WP1), extraction and analysis (WP2) of CHMs. While these fundamental issues are addressed, discussion forums emphasising the use of functional genomics methodology in research of the safety, efficacy and mechanisms of CHMs (WP3-WP7) and acupuncture (WP8) form the core of this coordination project. The project covers toxicology (WP3), *in vitro *and *in vivo *pharmacology (WP4-WP5), clinical studies (WP6), as well as international regulatory issues of CHM (WP7). WP9 is dedicated to organising the Final Conference of the consortium at the end of the project and WP10 is charged to manage consortium-wide matters, such as appointment and coordination of WP leadership, recruitment of additional experts, editing website and newsletters, drafting standard operating procedures, providing scientific and technological support and guidance, organising internal review and quality assurance, as well as liaising with the Commission and other stakeholders and external authorities.

### Membership

As shown in Table 1 the consortium has 27 beneficiary (*ie *funded) partner organisations across ten EU member states (*ie *Austria, Belgium, Estonia, Germany, Ireland, Italy, the Netherlands, Portugal, Spain and the UK) and China, which is an International Cooperation Partner Country (ICPC) of the EU. The consortium has additional 60 non-beneficiary (*ie *unfunded) collaborating partner organisations as well as two independent experts from Australia, Austria, Belgium, China, Denmark, Finland, Germany, Italy, Luxembourg, the Netherlands, Norway, Thailand, the UK and the USA (Table 2). Please refer to the project website at http://www.gp-tcm.org/about/partners/ for an updated list of consortium partners. This forms a diverse, multicultural and multidisciplinary team of about 150 principal investigators, including leading scientists, clinicians, TCM practitioners, as well as experts in industrial development and regulatory issues. Non-beneficiary membership is mainly based on consortium invitation and requires outstanding expertise needed for the project. Interested parties are welcome to contact us via the GP-TCM website http://www.gp-tcm.org/contact/. GP-TCM currently covers half of the 27 EU member states and its members in China are largely in major eastern and southern cities. As an open-ended consortium, GP-TCM welcomes interested parties from all EU member states and China to join our network, sharing resources and forging collaborations. We will continue to develop and strengthen collaborations with friends in Africa, South America, Asia and non-EU European countries to exchange experience and lessons learnt in the research of traditional medicines.

**Table 1:** GP-TCM beneficiary members

GP-TCM beneficiary partners	Country	Contact
King's College London	UK	Dr. Qihe Xu
University of Vienna	Austria	Prof. Verena M. Dirsch
L'Université Libre de Bruxelles	Belgium	Prof. Pierre Duez
Beijing University of Chinese Medicine	China	Prof Yanjiang Qiao
China Capital Medical University	China	Prof. Xiaomin Wang
Institute of Medicinal Plant Development	China	Prof. Xinmin Liu
Shanghai Institute of Acupuncture-Moxibustion	China	Prof. Huangan Wu
Shanghai University of Traditional Chinese Medicine	China	Dr. Liu Chenghai
University of Hong Kong	China	Dr. Hani El-Nezami
Asper Biotech Ltd	Estonia	Ms. Janne Üksti
Federal Institute for Drugs and Medical Devices	Germany	Dr. Werner Knöss
University of Düsseldorf	Germany	Prof. Peter Proksch
University of Munich	Germany	Prof. Angelika Vollmar
University of Bonn	Germany	Prof. Gabriele König
Trinity College Dublin	Ireland	Dr. Helen Sheridan
University of Milan	Italy	Prof. Enrica Bosisio
University of Padova	Italy	Prof. Maria Carrara
CMC Tasly Group BV	The Netherlands	Dr. You-Ping Zhu
Leiden University	The Netherlands	Prof. Robert Verpoorte
University of Minho	Portugal	Prof. Alberto Dias
University of Alcala	Spain	Prof. F. Javier de Lucio Cazaña
University Hospital Ramón y Cajal-FIBIO	Spain	Dr. M. Laura García Bermejo
Acu-herb Consultant, Sheffield	UK	Ms. Dan Jiang
University of Wolverhampton	UK	Prof. Kelvin Chan
Royal Botanic Gardens, Kew	UK	Prof. Monique Simmonds
University of Southampton	UK	Prof. George Lewith
University of Cambridge	UK	Dr. Tai-Ping Fan

**Table 2:** GP-TCM non-beneficiary members

GP-TCM Non-beneficiary partners	Country	Contact
University of Western Sydney	Australia	Prof. Alan Bensoussan
University of Graz	Austria	Prof. Rudolf Bauer
University of Mons	Belgium	Prof. Jean-Marie Colet
Beijing East Linden Science and Technology Co. Ltd.	China	Prof. Yanhuai Liu
China Academy of Chinese Medical Sciences	China	Prof. Aiping Lu
China-Japan Friendship Hospital	China	Prof. Ping Li
Dalian Institute of Chemical Physics	China	Prof. Xinmiao Liang
Hong Kong Baptist University	China	Prof. Zhongzhen Zhao
Hong Kong Buddhist Hospital	China	Prof. Vivian Wong
Jinan University	China	Prof. Xinsheng Yao
Peking University	China	Prof. Wenhan Lin
PuraPharm	China	Mr. Abraham Chan
Shanghai Innovation Research Centre of Traditional Medicine	China	Prof. William Weiguo Jia
Shanghai Jiaotong University	China	Prof. Liping Zhao
Shanghai Institute of Materia Medica, Chinese Academy of Sciences	China	Prof. De-an Guo
Tongji University	China	Prof. Gang Pei
Tasly Institute of Tasly Group Co., Ltd.	China	Ms. Karolina J. Svedlund
Tianjin University of Traditional Chinese Medicine	China	Prof. Boli Zhang
University of Macau	China	Prof. Yi-Tao Wang
The Chinese University of Hong Kong	China	Prof. Ge Lin
Pfizer Corporation Hong Kong Ltd	China	Mr. Stephen Leung
State Food and Drug Administration	China	Prof. Zhong-zhi Qian
National Research Institute of Chinese Medicine, Taiwan	China	Prof. Yi-Tsau Huang
University of Aarhus	Denmark	Prof. Brian Clark
University of Oulu	Finland	Prof. Olavi Pelkonen
Public Research Centre of Health	Luxembourg	Dr. Ning Wang
Charité University Medical Center	Germany	Prof. Claudia M. Witt
Johannes Gutenberg University	Germany	Dr. Huige Li
University of Regensburg	Germany	Prof. Gerhard Franz
Philipps - Universität Marburg	Germany	Prof. Shuming Li
Max Planck Institute for Biophysics	Germany	Prof. Wolfgang Schwarz
Dr Willmar Schwabe GmbH & Co. KG	Germany	Dr. Günter Meng
Caesar & Loretz GmbH	Germany	Dr. Mirko Bayer
University of Cagliari	Italy	Prof. Enzo Tramontano
University of Rome Tor Vergata	Italy	Prof. Giovanna M. Franconi
Institute of Neurobiology and Molecular Medicine, Italian National Research Council	Italy	Dr. Luigi Manni
SU BioMedicine	The Netherlands	Prof. Jan van der Greef
Norwegian University of Science and Technology	Norway	Prof. Odd Georg Nilsen
Thailand Ministry of Public Health	Thailand	Dr. Prat Boonyawongviroj
School of Pharmacy	UK	Prof. Michael Heinrich
Brunel University	UK	Prof. Ian A. Sutherland
University of Reading	UK	Prof. Elizabeth Williamson
Guy's & St Thomas' NHS Foundation Trust	UK	Prof. Debbie Shaw
University of Nottingham	UK	Prof. Sue Watson
University of Warwick	UK	Prof. Kenneth Muir
University of Westminster	UK	Dr. Volker Scheid
University of Lincoln	UK	Dr. Huijun Shen
Thames Valley University	UK	Prof. Nicola Robinson
University of the West England	UK	Prof. Quan Min Zhu
Imperial College London	UK	Dr. Daqing Ma
Global Regulatory Services	UK	Mrs. Greer Deal
Link China Pharma Solutions	UK	Mr. Marshall Ma
University of Oxford	UK	Ms. Rebecca Richmond
University of East London	UK	Dr. Tianjun Wang
Pharsafer Associates Limited	UK	Dr. Graeme Ladds
Avicenna	UK	Mr. Mazin Al-Khafaji
University of Louisville	USA	Prof. Y. James Kang
Yale University	USA	Prof. Yung-Chi Cheng
U.S. Food and Drug Administration	USA	Dr. Shaw T. Chen
Vanderbilt University Medical Center	USA	Dr. Lijun Ma
Independent	UK	Dr. Shouming Zhong
Independent	UK	Dr. Daryl Rees

### Progress and difficulties

During the first 18 months of the project (May 2009-October 2010), the management team (WP10) has coordinated a highly successful team build-up and re-construction. With the ever-strengthening expertise pool, WP10 has developed a number of committees and panels that lead the consortium with clear divisions of labour. WP10 has led the design and updates of the professional GP-TCM website and all WPs have established their homepages and online discussion facilities. Periodic newsletters have enabled members to share information and stay as a united team. Significantly, a series of face-to-face meetings, including consortium and WP kick-off meetings and the 1^st ^Annual General Meeting, have been organised to monitor the consortium, promote interactions and collaborations and ensure milestones are met and deliverables accomplished on time and in high quality.

Noteworthy WP-specific achievements are as follows. WP1 led the creation of a list of nearly 300 species of plants and fungi commonly used in TCM in Europe and China and a priority list of 11 species will be used by all WPs in their initial literature analysis. WP2 worked jointly with WP1, linking quality control, extraction technology and chemical analysis, with special emphasis on the important role of *paozhi *(processing) in the production of CHM. WP3 produced a list of toxic plants for further literature study and identified 3 major fields of action: (i) investigation of methods (classical and functional genomics) applicable to toxicity evaluation; (ii) study of toxicological reports available on a series of CHM; (iii) review of pharmacovigilance safety data. WP4 established evaluation criteria for scoring scientific articles and began the creation of an appropriate database of literature encompassing functional genomic applications in CHM research. WP5 performed reviews on CHM literature involving animal models, especially models of cancer and its conclusions have laid a solid foundation for further literature analysis on application of functional genomics in CHM research and proposing good practice in animal studies of CHM. WP6 gathered literature on seminal studies in clinical CHM studies and drafted a guideline on clinical trials of CHM. WP6 and WP8 collaboratively designed an online survey targeting TCM practitioners and the survey is currently undergoing in collaboration with 30 professional acupuncture and TCM organisations. WP7 brought together wide-ranging experiences and expertise in drug development and registration from Europe, China, Australia and North America to discuss the legislative and regulatory issues relevant to CHM. Together they are developing a comprehensive document providing comparisons of different practices on CHM regulations and this will be extremely helpful for the EU to develop its future policies and for companies to develop products for the global market. WP9 discussed the time and format of the Final Conference and preliminary bookings of venue has been made.

The major difficulty encountered by the consortium is unsurprisingly the language barrier. There is a lack of accessibility to original Chinese publications in Europe, and even if they are available, fast and accurate translation of these materials is impossible, preventing the consortium from extensively studying classic Chinese medical literature and evaluating a great deal of modern Chinese medical literature. While we strongly encourage our members to master both English and Chinese languages, we welcome members from various linguistic, ethnic and cultural backgrounds to work in close collaboration.

### Further work

As the first ever EU-China collaborative consortium dedicated to TCM research, we will continue to promote EU-China dialogues and collaborations in this important emerging supra-disciplinary area. As a network of principal investigators, we will collaborate to train the next generation of scientists who are more comprehensively equipped to study complex drugs such as CHM and personalised medicine such as TCM. As a coordination action involving much literature review and evaluation, we acknowledge the huge importance of good practice in scientific publication and will continue to support open-access publications.

### Concluding remarks

As an EU-China collaboration dedicated to TCM research, we are keen to incorporate ourselves into the worldwide landscape of TCM research community and serve as a constructive member. We sincerely support the international TCM community to bundle forces to improve TCM research funding from both the public and private sectors and to help shape the medicine of tomorrow together.

## Abbreviations

CGCM: Consortium for Globalisation of Chinese Medicine; CHM: Chinese herbal medicines; EU: European Union; FP7: Seventh Framework Programme; GP-TCM: Good Practice in Traditional Chinese Medicine Research in the Post-genomic Era; ICPC: International Cooperation Partner Country; ISCM: International Society for Chinese Medicine; TCM: Traditional Chinese medicine; WFCMS: World Federation of Chinese Medicine Societies; WPs: work packages.

## Competing interests

The authors declare that they have no competing interests.

## Authors' contributions

The authors are the Project Manager (HU), the Coordinator (QX), Deputy Coordinators (AD, DG, TPF) and Assistant Coordinator (HE) of the GP-TCM Consortium. HU and QX jointly drafted the manuscript. All named authors took part in the revision and approved the final version of the paper.
